# The Distribution of Different Types of Diabetes in Childhood: A Single Center Experience

**DOI:** 10.4274/jcrpe.5204

**Published:** 2018-05-18

**Authors:** Belma Haliloğlu, Saygın Abalı, Fuat Buğrul, Enes Çelik, Serpil Baş, Zeynep Atay, Tülay Güran, Serap Turan, Abdullah Bereket

**Affiliations:** 1 Yeditepe University Faculty of Medicine, Department of Pediatric Endocrinology, İstanbul, Turkey; 2 Acibadem University Faculty of Medicine, Department of Pediatric Endocrinology, İstanbul, Turkey; 3 Marmara University Faculty of Medicine, Department of Pediatric Endocrinology, İstanbul, Turkey; 4 Necip Fazıl City Hospital, Clinic of Pediatric Endocrinology, Kahramanmaraş, Turkey; 5 Medipol University Faculty of Medicine, Department of Pediatric Endocrinology, İstanbul, Turkey

**Keywords:** Type 1 diabetes, type 2 diabetes, MODY, childhood

## Abstract

**Objective:**

Type 1 diabetes (T1D) is the most common cause of diabetes in childhood but type 2 diabetes (T2D) and maturity onset diabetes of the young (MODY) are emerging as noteworthy causes of diabetes at young ages. The aim is to determine the distribution, trends and clinical features of the different types of diabetes in childhood in one tertiary center.

**Methods:**

The records of children and adolescents aged 0-18 years who were diagnosed as “diabetes/persistent hyperglycemia” between January 1999 and December 2016, were reviewed. Clinical and laboratory characteristics of the patients at diagnosis and type of diabetes were recorded.

**Results:**

Children and adolescents aged 0-18 years who were diagnosed "diabetes/persistent hyperglycemia" between January 1999 and December 2016, were reviewed. Clinical and laboratory characteristics of the patients at the diagnosis and type of diabetes were recorded.The mean ± standard deviation age of 835 patients (48.7% females) at diagnosis was 8.8±4.4 years. Eighty-four percent of the patients were diagnosed as T1D, 5.7% as T2D, 5.3% as clinical MODY and 5% as being cases of other types of diabetes. The frequency of diabetic ketoacidosis (DKA) and severe DKA in T1D were 48.4% and 11.6%, respectively. Fourteen patients (29.2%) with T2D presented with ketosis and two of them (4.2%) had DKA at diagnosis. Antibody positivity was 83.1% in T1D and 14.8% in T2D. A statistically significant increase in the frequency of T2D has clearly been demonstrated in recent years with a frequency of 1.9%, 2.4% and 7.9% in 1999-2004, 2005-2010 and 2011-2016, respectively (p<0.001). In MODY, genetic analysis was performed in 26 (59%) patients and HNF1A and GCK gene mutations were detected in 3 (11.5%) and 14 (53.8%) patients, respectively.

**Conclusion:**

Although the most frequent cause of DM is T1D in childhood, a trend towards increase in the frequency of T2D in recent years is notable in our population.

## What is already known on this topic?

Although type 1 diabetes is the most common type of diabetes in childhood, a variable increase in the prevalence of type 2 diabetes  and maturity onset diabetes of the young has been reported by different multicenter studies depending on the ethnic background, the country of residence and the availability of genetic tests.

## What this study adds?

Information on the distribution of type of diabetes in the Turkish pediatric population is scarce. Comparative data on the clinical characteristics of different types of diabetes, based on the experience of a tertiary pediatric diabetes center over the last 17 years, are presented in this paper. Also, this paper identified a trend towards increase in the frequency of  type 2 diabetes  in Turkish pediatric population.

## Introduction

Type 1 diabetes (T1D) is the most common type of diabetes in childhood and its incidence is still rising in various parts of the world (1). However, the increasing worldwide rates of child obesity have also been associated with a variable increase in the prevalence of type 2 diabetes (T2D), depending on the ethnic background and the country of residence (2). While the prevalence of T2D in children was reported as 11% in the USA (3), this ratio was reported to be lower in Europe (1.3% in SWEET) (4).

Childhood T2D can be confused with maturity onset diabetes of the young (MODY) due to the presence of a family history, presenting features and a possible confounding factor of obesity/overweight (5,6). Furthermore, MODY, especially due to HNF1A mutations can be misclassified as T1D (7). Determining the type of diabetes is important for therapeutic considerations as well as genetic counseling (8).

The aims of the present study were: 1) to review the etiologic distribution and temporal changes in the etiology of childhood diabetes and; 2) to compare the clinical characteristics of the different types of diabetes encountered in a tertiary pediatric diabetes center over the last 17 years.

## Methods

Data on 927 children and adolescents aged <18 years who were diagnosed as “diabetes” or “persistent hyperglycemia” and who were followed-up at the Pediatric Endocrinology and Diabetes Unit of Marmara University Faculty of Medicine in İstanbul, Turkey, between January 1999 and December 2016, were examined. Ninety-two patients with a follow up duration of less than one year were excluded, since the type of diabetes could not be specified because of insufficient data. Finally, 835 patients were included in this single-centered, observational, retrospective study. 

The patients’ gender, age of diagnosis, height (cm), weight (kg), body mass index (BMI, kg/m^2^), c-peptide level (ng/mL), presence of pancreatic autoantibodies (islet cell antibodies, glutamic acid decarboxylase antibodies and insulin autoantibodies), presence of ketone bodies, pH and HCO_3_ levels at the time of diagnosis, type of diabetes, treatment modalities (diet, oral antidiabetic drug, insulin) were recorded. The patients were classified according to the ISPAD Consensus 2014 (Table 1).

T1D was diagnosed in the presence of severe insulin deficiency, autoantibody positivity and the absence of any suggestive signs of other causes of diabetes. The diagnostic criteria for T2D were based on overweight/obesity, clinical findings of insulin resistance (acanthosis nigricans, hypertension, dyslipidemia), family history of T2D and good metabolic control with metformin or metformin combined with low dose, long-acting insulin (<0.5 U/kg/d). Patients who had a family history of diabetes of at least two generations in one side of the family, negative autoantibodies, no evidence of insulin resistance and good metabolic control with diet, sulphonylurea or low dose insulin were classified as clinically MODY. *HNF1A, HNF4A *and *GCK* genes were analysed for clinically suspected MODY cases. Children with an onset of diabetes before six months of age were diagnosed as neonatal diabetes mellitus (NDM) and relevant genetic tests were performed. 

The study was approved by the local Ethical Committee of Marmara University (approval no: 09.2013.0408).

### Statistical Analysis

All statistical data were analyzed using SPSS statistical software for Windows, version 17.0 (SPSS, Chicago, IL). Variables were summarized with descriptive statistics. Data were presented as mean ± standard deviation (SD). Normality was assessed using the Kolmogorov-Smirnov test. Parametric and nonparametric tests were used for inter-group comparisons. Chi-square test was used for categorical variables. Student’s t-test was applied for continuous variables in independent groups. The Mann-Whitney U test was used for continuous variables that did not show normal distribution. The level of statistical significance was set as p=0.05.

## Results

After 92/927 patients (9.9%) were excluded, the mean age of 835 patients (48.7% females) at diagnosis was 8.8±4.4 (median 9.0, range 0.0-18.0) years. Seven hundred and one patients were diagnosed with T1D (84%), 48 with T2D (5.7%), 44 with clinical MODY (5.3%) and 42 with other types of diabetes (5%) (Table 1). 

The clinical characteristics at diagnosis of T1D, T2D and MODY are shown in Table 2. In T1D, 23.7% (n=166) were younger than age 5 years and 1.6% (n=8) had a BMI standard deviation score (SDS) >2. The frequency of severe diabetic ketoacidosis (DKA) at diagnosis in T1D was 11.6%. T2D was more common in girls and older children. Fourteen patients with T2D (29.2%) presented with ketosis and two of these (4.2%) had DKA at diagnosis. Diabetes autoantibody positivity was 83.1% in T1D and 14.8% in T2D. The patients with antibody-positive T2D were compared with those with antibody-negative T2D in terms of age, BMI SDS, presence of DKA and use of insulin. The only statistically significant difference was age at diagnosis. Antibody-positive T2D patients were younger than the antibody negative (11.8±3.3 vs 13.7±1.94, p=0.045) patients.

**Figure 1:**
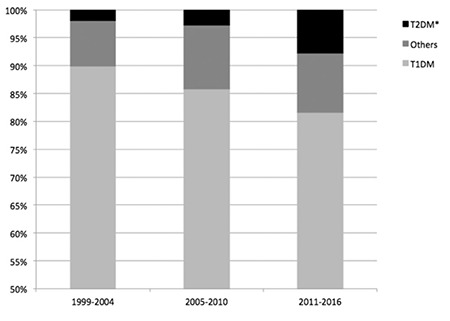
Frequency of type of diabetes in a single-center by 6 year periods T1DM: type 1 diabetes mellitus, *: p<0.05, T2DM: type 2 diabetes mellitus

A statistically significant increase in the frequency of T2D has clearly been demonstrated in recent years in our cohort with a frequency of 1.9%, 2.4% and 7.9% in the time periods 1999-2004, 2005-2010 and 2011-2016, respectively (p<0.001) (Figure 1).

The frequency of DKA was 58.4% and there was also a statistically significant decrease in the proportion of ketoacidosis at diagnosis in T1D after year 2011 (55% vs 44.6%, p=0.022). However, the decrement in the proportion of severe ketoacidosis was not statistically significant (15.1% vs 9.7%, p=0.066). 

Mean c-peptide levels at diagnosis were 0.7±0.6 ng/mL in T1D, 3.2±1.5 ng/mL in T2D and 1.3±0.6 ng/mL in MODY patients (p<0.001) (Table 2).

In MODY, genetic analysis was available in 26 (59%) patients and *HNF1A* and *GCK* gene mutations were detected in 3 (11.5%) and 14 (53.8%) patients, respectively. 

Seven patients had NDM and it was molecularly confirmed in 6 of 7 patients in whom *KCNJ11 *(n=2), *6q24* (n=1), *EIF2AK3* (n=1), *SLC19A2* (n=1) and *PTF1A *enhancer (n=1) gene mutations were identified. 

In eight (2F/6M) patients with Wolfram syndrome from five families, three known homozygote/compound heterozygote mutations in *WFS* gene were detected and four of them had optic atrophy, one had cataract and one had diabetes insipidus.

Cystic fibrosis-related diabetes (CFRD) was detected in 11 patients (6F/5M, 1.3%) and the mean age and mean BMI SDS at diagnosis were 12.7±4.1 (5.0-17.4) and -1.4±1.5 (-3.7-1.1), respectively.

The frequency of drug-induced diabetes was 0.6% (n=5), four of which were due to L-asparaginase and one due to tacrolimus.

## Discussion

The present study illuminated some issues concerning the frequency of the different types of diabetes in our population and allowed us to make comparisons with other societies. The overall frequency of T1D, T2D, MODY and other specific types of diabetes were 84%, 5.7%, 5.3% and 5%, respectively. 

T1D is still the most common cause of childhood diabetes and its frequency varies between 85-95% in different regions of the world (3,4,7). This variability originates from the number of children with T2D and MODY. The frequencies of T1D, T2D and MODY were 85.6%, 10.8% and 1.2% respectively in the SEARCH study (USA), while these ratios were 95.5%, 1.3% and 1.5% respectively in the SWEET study (Europe) (3,4,9). Also, the frequency of MODY was higher (5.5%) in a recent study from Italy (7). The variation in the frequencies could be explained by the availability of genetic testing and also by prevalence of obesity in that region. Misclassification of diabetes due to the lack of evidence-based clinical criteria for differential diagnosis is widespread and reported to be 7-15% (10). The diagnosis of MODY (5.3%) and T2D (5.7%) was found to be more common in this study as compared to the SWEET study. The present study is not a national multicenter study, so this difference may be explained by referral of the rare types of diabetes to our tertiary center. 

The most confusing factor for classification of diabetes is obesity. BMI at the time of diagnosis is a less discriminatory feature for classification (10), since the increase in obesity has led to the appearance of children with obese T1D/MODY. In different studies, the frequency of obesity among patients with T1D at the time of diagnosis was 3.1-9% (11,12), but it was 1.6% in the present study. This could be due to lower obesity rates in our pediatric population (13) compared to North America and Western Europe. Although lower than these regions, obesity rates are also increasing in Turkey which may be the reason for the increase in the frequency of T2D observed over the time span of this study, from 1.9% to 7.9%. In accordance with previous reports (14), T2D was more common in girls and at pubertal ages. 

The antibody positivity in T2D is reported up to 15% and these antibody-positive patients are usually younger, less overweight/obese and have higher hemoglobin A1c values (15). So, several terminologies have been recommended such as double diabetes, type 1.5 diabetes and latent autoimmune diabetes of youth. In the present study, the antibody positivity was 14.8% and there was a significant difference between antibody positive and negative subjects only at the age of diagnosis, with a younger age of diagnosis being seen in antibody positive patients, in line with other reports. Although, a few case reports described antibody positive MODY patients, the prevalence of antibody positivity in MODY is <1% (16). Therefore, the antibody positivity was used as an exclusion criterion for MODY in the present study.

The frequency of DKA in T1D varied from 48% to 66% in the different studies in Turkey (17,18,19). Our study shows a decrease of 10% in the rate of DKA at the time of diagnosis, albeit, the current ratio is still high. DKA at the time of diagnosis of pediatric T2D is not infrequent and is reported to be as high as 40% of patients (15). However, it was not frequent in our study (4.2%) but nearly one-third of patients with T2D presented with ketosis without acidosis.

The frequency of MODY varies between 0.83-5.5% in different studies (4,6,7,20,21,22,23). GCK mutation (up to 95%) was the most common cause in the studies that reported higher MODY frequency (6,7,22). Similarly, we detected GCK mutation in 53.8% of the clinically MODY patients who were genetically tested. This can be explained by the widespread use of random glucose measurement in general pediatric clinics in Turkey. On the other hand, the rate of genetic analysis in the clinical MODY patients was low (59%) in the present study, as it was not possible to perform this analysis prior to 2010. In 65.3% of these patients a mutation in one of the known MODY genes could be detected. This ratio varies between 27-89% in different studies (24). This variation and failure to detect mutations may result from inclusion criteria for genetic testing, may be due to a mutation in a gene not yet identified or to diagnostic overlap of different types of diabetes. 

C peptide levels, although useful in long-standing diabetes cases, might not be discriminative in patients with new onset diabetes because of substantial overlap among different types of diabetes mellitus (25). Nevertheless, in addition to autoantibody positivity, c-peptide levels remain a relatively good diagnostic parameter. In the present study, c-peptide levels at the time of diagnosis were helpful, especially in differentiating between T1D and T2D.

### Study Limitations

The limitation of this study is that it included a tertiary center data. Therefore, the frequency of some specific types of diabetes as CFRD may not reflect real frequency.

## Conclusion

The present study provides trends over the last 17 years in pediatric diabetes in a large number of patients, from a single tertiary center and tries to identify the distinguishing features of each of different types of diabetes. The frequency of T2D is increasing but is still lower than that in North America. MODY is becoming more easily recognized in recent years owing to availability of autoantibody testing and genetic tests. Despite overlapping features such as obesity, ketosis and antibody positivity, there are demographic (age, puberty, gender, family history) as well as laboratory (autoantibody positivity, c-peptide) tools to correctly identify the type of diabetes in the pediatric population.

## Figures and Tables

**Table 1:**
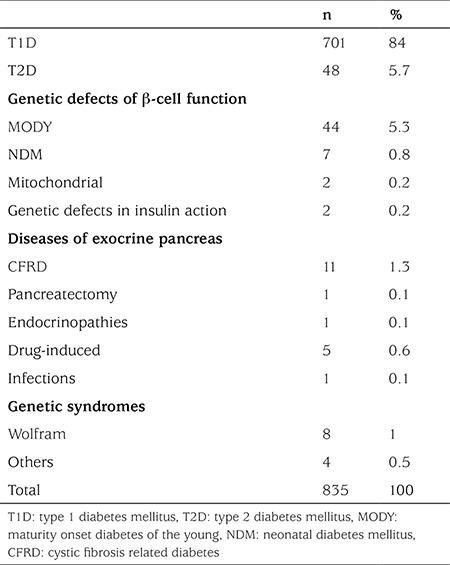
The distribution of the patients with diabetes

**Table 2:**
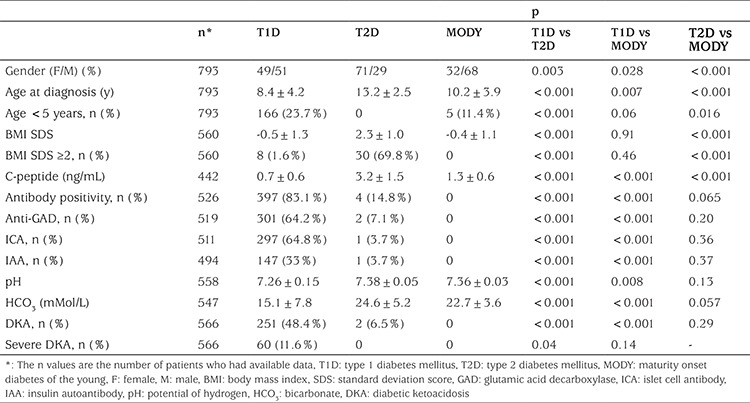
The clinical features of the patients with type 1 diabetes mellitus, type 2 diabetes mellitus and maturity onset diabetes of the young at diagnosis
